# CARD 2023: expanded curation, support for machine learning, and resistome prediction at the Comprehensive Antibiotic Resistance Database

**DOI:** 10.1093/nar/gkac920

**Published:** 2022-10-20

**Authors:** Brian P Alcock, William Huynh, Romeo Chalil, Keaton W Smith, Amogelang R Raphenya, Mateusz A Wlodarski, Arman Edalatmand, Aaron Petkau, Sohaib A Syed, Kara K Tsang, Sheridan J C Baker, Mugdha Dave, Madeline C McCarthy, Karyn M Mukiri, Jalees A Nasir, Bahar Golbon, Hamna Imtiaz, Xingjian Jiang, Komal Kaur, Megan Kwong, Zi Cheng Liang, Keyu C Niu, Prabakar Shan, Jasmine Y J Yang, Kristen L Gray, Gemma R Hoad, Baofeng Jia, Timsy Bhando, Lindsey A Carfrae, Maya A Farha, Shawn French, Rodion Gordzevich, Kenneth Rachwalski, Megan M Tu, Emily Bordeleau, Damion Dooley, Emma Griffiths, Haley L Zubyk, Eric D Brown, Finlay Maguire, Robert G Beiko, William W L Hsiao, Fiona S L Brinkman, Gary Van Domselaar, Andrew G McArthur

**Affiliations:** David Braley Centre for Antibiotic Discovery, McMaster University, Hamilton, Ontario, Canada; Michael G. DeGroote Institute for Infectious Disease Research, McMaster University, Hamilton, Ontario, Canada; Department of Biochemistry and Biomedical Sciences, McMaster University, Hamilton, Ontario, Canada; David Braley Centre for Antibiotic Discovery, McMaster University, Hamilton, Ontario, Canada; Michael G. DeGroote Institute for Infectious Disease Research, McMaster University, Hamilton, Ontario, Canada; Department of Biochemistry and Biomedical Sciences, McMaster University, Hamilton, Ontario, Canada; David Braley Centre for Antibiotic Discovery, McMaster University, Hamilton, Ontario, Canada; Michael G. DeGroote Institute for Infectious Disease Research, McMaster University, Hamilton, Ontario, Canada; Department of Biochemistry and Biomedical Sciences, McMaster University, Hamilton, Ontario, Canada; David Braley Centre for Antibiotic Discovery, McMaster University, Hamilton, Ontario, Canada; Michael G. DeGroote Institute for Infectious Disease Research, McMaster University, Hamilton, Ontario, Canada; Department of Biochemistry and Biomedical Sciences, McMaster University, Hamilton, Ontario, Canada; David Braley Centre for Antibiotic Discovery, McMaster University, Hamilton, Ontario, Canada; Michael G. DeGroote Institute for Infectious Disease Research, McMaster University, Hamilton, Ontario, Canada; Department of Biochemistry and Biomedical Sciences, McMaster University, Hamilton, Ontario, Canada; David Braley Centre for Antibiotic Discovery, McMaster University, Hamilton, Ontario, Canada; Michael G. DeGroote Institute for Infectious Disease Research, McMaster University, Hamilton, Ontario, Canada; Department of Biochemistry and Biomedical Sciences, McMaster University, Hamilton, Ontario, Canada; David Braley Centre for Antibiotic Discovery, McMaster University, Hamilton, Ontario, Canada; Michael G. DeGroote Institute for Infectious Disease Research, McMaster University, Hamilton, Ontario, Canada; Department of Biochemistry and Biomedical Sciences, McMaster University, Hamilton, Ontario, Canada; Department of Computer Science, University of Manitoba, Winnipeg, Manitoba, Canada; National Microbiology Laboratory, Public Health Agency of Canada, Winnipeg, Manitoba, Canada; David Braley Centre for Antibiotic Discovery, McMaster University, Hamilton, Ontario, Canada; Michael G. DeGroote Institute for Infectious Disease Research, McMaster University, Hamilton, Ontario, Canada; Department of Biochemistry and Biomedical Sciences, McMaster University, Hamilton, Ontario, Canada; David Braley Centre for Antibiotic Discovery, McMaster University, Hamilton, Ontario, Canada; Michael G. DeGroote Institute for Infectious Disease Research, McMaster University, Hamilton, Ontario, Canada; Department of Biochemistry and Biomedical Sciences, McMaster University, Hamilton, Ontario, Canada; David Braley Centre for Antibiotic Discovery, McMaster University, Hamilton, Ontario, Canada; Michael G. DeGroote Institute for Infectious Disease Research, McMaster University, Hamilton, Ontario, Canada; Department of Biochemistry and Biomedical Sciences, McMaster University, Hamilton, Ontario, Canada; David Braley Centre for Antibiotic Discovery, McMaster University, Hamilton, Ontario, Canada; Michael G. DeGroote Institute for Infectious Disease Research, McMaster University, Hamilton, Ontario, Canada; Department of Biochemistry and Biomedical Sciences, McMaster University, Hamilton, Ontario, Canada; David Braley Centre for Antibiotic Discovery, McMaster University, Hamilton, Ontario, Canada; Michael G. DeGroote Institute for Infectious Disease Research, McMaster University, Hamilton, Ontario, Canada; Department of Biochemistry and Biomedical Sciences, McMaster University, Hamilton, Ontario, Canada; David Braley Centre for Antibiotic Discovery, McMaster University, Hamilton, Ontario, Canada; Michael G. DeGroote Institute for Infectious Disease Research, McMaster University, Hamilton, Ontario, Canada; Department of Biochemistry and Biomedical Sciences, McMaster University, Hamilton, Ontario, Canada; David Braley Centre for Antibiotic Discovery, McMaster University, Hamilton, Ontario, Canada; Michael G. DeGroote Institute for Infectious Disease Research, McMaster University, Hamilton, Ontario, Canada; Department of Biochemistry and Biomedical Sciences, McMaster University, Hamilton, Ontario, Canada; David Braley Centre for Antibiotic Discovery, McMaster University, Hamilton, Ontario, Canada; Michael G. DeGroote Institute for Infectious Disease Research, McMaster University, Hamilton, Ontario, Canada; Department of Biochemistry and Biomedical Sciences, McMaster University, Hamilton, Ontario, Canada; David Braley Centre for Antibiotic Discovery, McMaster University, Hamilton, Ontario, Canada; Michael G. DeGroote Institute for Infectious Disease Research, McMaster University, Hamilton, Ontario, Canada; Department of Biochemistry and Biomedical Sciences, McMaster University, Hamilton, Ontario, Canada; David Braley Centre for Antibiotic Discovery, McMaster University, Hamilton, Ontario, Canada; Michael G. DeGroote Institute for Infectious Disease Research, McMaster University, Hamilton, Ontario, Canada; Department of Biochemistry and Biomedical Sciences, McMaster University, Hamilton, Ontario, Canada; David Braley Centre for Antibiotic Discovery, McMaster University, Hamilton, Ontario, Canada; Michael G. DeGroote Institute for Infectious Disease Research, McMaster University, Hamilton, Ontario, Canada; Department of Biochemistry and Biomedical Sciences, McMaster University, Hamilton, Ontario, Canada; David Braley Centre for Antibiotic Discovery, McMaster University, Hamilton, Ontario, Canada; Michael G. DeGroote Institute for Infectious Disease Research, McMaster University, Hamilton, Ontario, Canada; Department of Biochemistry and Biomedical Sciences, McMaster University, Hamilton, Ontario, Canada; David Braley Centre for Antibiotic Discovery, McMaster University, Hamilton, Ontario, Canada; Michael G. DeGroote Institute for Infectious Disease Research, McMaster University, Hamilton, Ontario, Canada; Department of Biochemistry and Biomedical Sciences, McMaster University, Hamilton, Ontario, Canada; David Braley Centre for Antibiotic Discovery, McMaster University, Hamilton, Ontario, Canada; Michael G. DeGroote Institute for Infectious Disease Research, McMaster University, Hamilton, Ontario, Canada; Department of Biochemistry and Biomedical Sciences, McMaster University, Hamilton, Ontario, Canada; David Braley Centre for Antibiotic Discovery, McMaster University, Hamilton, Ontario, Canada; Michael G. DeGroote Institute for Infectious Disease Research, McMaster University, Hamilton, Ontario, Canada; Department of Biochemistry and Biomedical Sciences, McMaster University, Hamilton, Ontario, Canada; David Braley Centre for Antibiotic Discovery, McMaster University, Hamilton, Ontario, Canada; Michael G. DeGroote Institute for Infectious Disease Research, McMaster University, Hamilton, Ontario, Canada; Department of Biochemistry and Biomedical Sciences, McMaster University, Hamilton, Ontario, Canada; Department of Molecular Biology and Biochemistry, Simon Fraser University, Burnaby, British Columbia, Canada; Research Computing Group, Simon Fraser University, Burnaby, British Columbia, Canada; Department of Molecular Biology and Biochemistry, Simon Fraser University, Burnaby, British Columbia, Canada; David Braley Centre for Antibiotic Discovery, McMaster University, Hamilton, Ontario, Canada; Michael G. DeGroote Institute for Infectious Disease Research, McMaster University, Hamilton, Ontario, Canada; Department of Biochemistry and Biomedical Sciences, McMaster University, Hamilton, Ontario, Canada; David Braley Centre for Antibiotic Discovery, McMaster University, Hamilton, Ontario, Canada; Michael G. DeGroote Institute for Infectious Disease Research, McMaster University, Hamilton, Ontario, Canada; Department of Biochemistry and Biomedical Sciences, McMaster University, Hamilton, Ontario, Canada; David Braley Centre for Antibiotic Discovery, McMaster University, Hamilton, Ontario, Canada; Michael G. DeGroote Institute for Infectious Disease Research, McMaster University, Hamilton, Ontario, Canada; Department of Biochemistry and Biomedical Sciences, McMaster University, Hamilton, Ontario, Canada; David Braley Centre for Antibiotic Discovery, McMaster University, Hamilton, Ontario, Canada; Michael G. DeGroote Institute for Infectious Disease Research, McMaster University, Hamilton, Ontario, Canada; Department of Biochemistry and Biomedical Sciences, McMaster University, Hamilton, Ontario, Canada; David Braley Centre for Antibiotic Discovery, McMaster University, Hamilton, Ontario, Canada; Michael G. DeGroote Institute for Infectious Disease Research, McMaster University, Hamilton, Ontario, Canada; Department of Biochemistry and Biomedical Sciences, McMaster University, Hamilton, Ontario, Canada; David Braley Centre for Antibiotic Discovery, McMaster University, Hamilton, Ontario, Canada; Michael G. DeGroote Institute for Infectious Disease Research, McMaster University, Hamilton, Ontario, Canada; Department of Biochemistry and Biomedical Sciences, McMaster University, Hamilton, Ontario, Canada; David Braley Centre for Antibiotic Discovery, McMaster University, Hamilton, Ontario, Canada; Michael G. DeGroote Institute for Infectious Disease Research, McMaster University, Hamilton, Ontario, Canada; Department of Biochemistry and Biomedical Sciences, McMaster University, Hamilton, Ontario, Canada; David Braley Centre for Antibiotic Discovery, McMaster University, Hamilton, Ontario, Canada; Michael G. DeGroote Institute for Infectious Disease Research, McMaster University, Hamilton, Ontario, Canada; Department of Biochemistry and Biomedical Sciences, McMaster University, Hamilton, Ontario, Canada; Faculty of Health Sciences, Simon Fraser University, Burnaby, British Columbia, Canada; Faculty of Health Sciences, Simon Fraser University, Burnaby, British Columbia, Canada; David Braley Centre for Antibiotic Discovery, McMaster University, Hamilton, Ontario, Canada; Michael G. DeGroote Institute for Infectious Disease Research, McMaster University, Hamilton, Ontario, Canada; Department of Biochemistry and Biomedical Sciences, McMaster University, Hamilton, Ontario, Canada; David Braley Centre for Antibiotic Discovery, McMaster University, Hamilton, Ontario, Canada; Michael G. DeGroote Institute for Infectious Disease Research, McMaster University, Hamilton, Ontario, Canada; Department of Biochemistry and Biomedical Sciences, McMaster University, Hamilton, Ontario, Canada; Faculty of Computer Science, Dalhousie University, Halifax, Nova Scotia, Canada; Institute for Comparative Genomics, Dalhousie University, Halifax, Nova Scotia, Canada; Department of Community Health & Epidemiology, Dalhousie University, Halifax, Nova Scotia, Canada; Faculty of Computer Science, Dalhousie University, Halifax, Nova Scotia, Canada; Institute for Comparative Genomics, Dalhousie University, Halifax, Nova Scotia, Canada; Department of Molecular Biology and Biochemistry, Simon Fraser University, Burnaby, British Columbia, Canada; Faculty of Health Sciences, Simon Fraser University, Burnaby, British Columbia, Canada; Department of Molecular Biology and Biochemistry, Simon Fraser University, Burnaby, British Columbia, Canada; National Microbiology Laboratory, Public Health Agency of Canada, Winnipeg, Manitoba, Canada; Department of Medical Microbiology and Infectious Diseases, Max Rady College of Medicine, University of Manitoba, Winnipeg, Manitoba, Canada; David Braley Centre for Antibiotic Discovery, McMaster University, Hamilton, Ontario, Canada; Michael G. DeGroote Institute for Infectious Disease Research, McMaster University, Hamilton, Ontario, Canada; Department of Biochemistry and Biomedical Sciences, McMaster University, Hamilton, Ontario, Canada

## Abstract

The Comprehensive Antibiotic Resistance Database (CARD; card.mcmaster.ca) combines the Antibiotic Resistance Ontology (ARO) with curated AMR gene (ARG) sequences and resistance-conferring mutations to provide an informatics framework for annotation and interpretation of resistomes. As of version 3.2.4, CARD encompasses 6627 ontology terms, 5010 reference sequences, 1933 mutations, 3004 publications, and 5057 AMR detection models that can be used by the accompanying Resistance Gene Identifier (RGI) software to annotate genomic or metagenomic sequences. Focused curation enhancements since 2020 include expanded β-lactamase curation, incorporation of likelihood-based AMR mutations for *Mycobacterium tuberculosis*, addition of disinfectants and antiseptics plus their associated ARGs, and systematic curation of resistance-modifying agents. This expanded curation includes 180 new AMR gene families, 15 new drug classes, 1 new resistance mechanism, and two new ontological relationships: *evolutionary_variant_of* and *is_small_molecule_inhibitor*. *In silico* prediction of resistomes and prevalence statistics of ARGs has been expanded to 377 pathogens, 21,079 chromosomes, 2,662 genomic islands, 41,828 plasmids and 155,606 whole-genome shotgun assemblies, resulting in collation of 322,710 unique ARG allele sequences. New features include the CARD:Live collection of community submitted isolate resistome data and the introduction of standardized 15 character CARD Short Names for ARGs to support machine learning efforts.

## INTRODUCTION

In the decade since the initial publication of the Comprehensive Antibiotic Resistance Database (CARD) (1), the public health threat of antimicrobial resistance (AMR) has grown significantly, prompting a collective global initiative to combat the spread of AMR through collaborative research, molecular and phenotypic surveillance, and antimicrobial stewardship (2–5). However, the complexity and global burden of the AMR crisis (6) continues to expand with the continuous discovery of novel antibiotic resistance genes (ARGs), including those using novel mechanisms such as MCR-mediated colistin resistance via change in the charge of the cell membrane (7) or ejection of rifamycin from its target RNA polymerase by helicase-like protein HelR (8), as well as contributing factors from environmental, veterinary, and food-related sources (9,10). Furthermore, emerging evidence details a complex relationship between AMR and the COVID-19 pandemic, in part due to prolonged hospitalizations, relaxed antibiotic stewardship in overwhelmed hospital settings, and increased household and topical biocide use (11,12).

As previously described (13), CARD is an ontology-centric knowledgebase on the molecular determinants of antibiotic resistance. By integrating a detailed controlled vocabulary, the Antibiotic Resistance Ontology (ARO), with ARG molecular sequences and mutation data, CARD is able to provide both reference set and software tools for guiding AMR research, particularly for ARG annotation and discovery from genomic and metagenomic data. One such analytical framework is the Resistance Gene Identifier (RGI), CARD’s algorithm for computational AMR genotype and phenotype prediction from genomic data using the bioinformatics detection models curated in CARD. To ensure accuracy, CARD is manually curated by a team of biocurators and researchers, following a core curation paradigm. Additionally, CARD works towards harmonization with other AMR databases, specifically including those provided by the National Center for Biotechnology Information (NCBI), such as the Pathogen Detection Reference Gene Catalog (https://www.ncbi.nlm.nih.gov/pathogens/).

Here we provide an update on the state of CARD and the ARO, with a review of the available data and updates since our 2020 publication. We discuss in detail (i) general and specific improvements to the ARO and curation of AMR in CARD, (ii) improved integration and harmonization with existing resources for specific AMR gene families (such as β-lactamase resistance genes) and pathogens (such as *Mycobacterium tuberculosis*), (iii) curation of resistance modifying agents, (iv) gene name standardization in support of machine learning efforts, (v) improvements to CARD’s Model Ontology in support of bioinformatic annotation of genomes or metagenomes, (vi) description of CARD’s curation paradigms, protocols and quality assurance algorithms and (vii) expansion of the previously introduced CARD Resistomes & Variants (CARD-R) resource.

## EXPANSION OF CARD CURATION

### Current state of CARD and the ARO

CARD’s primary curation paradigm continues as previously described: ‘to be included in CARD an AMR determinant must be described in a peer-reviewed scientific publication, with its DNA sequence available in GenBank, including clear experimental evidence of elevated minimum inhibitory concentration (MIC) over controls. AMR genes predicted by *in silico* methods, but not experimentally characterized, are not included in CARD’s primary curation’ (13). Data in CARD is organized using four core ontologies: the ARO describing all aspects of AMR, the CARD Model Ontology (MO) defining bioinformatics parameters for annotating ARGs in genomic data, the CARD Relations Ontology (RO) for association of terms within ontologies, and NCBITaxon for tracking the source organisms and taxonomic distribution of ARG sequences. The ARO is the primary ontology for organization and interpretation of data within CARD and it contains seven major branches: antibiotic molecule (ARO:1000003), mechanism of antibiotic resistance (ARO:1000002), determinant of antibiotic resistance (ARO:3000000), resistance-modifying agents (ARO:0000076), antibiotic target (ARO:3000708), antibiotic biosynthesis (ARO:3000082), and component of AMR genotypic or phenotypic terminology (ARO:3000045). Each curated ARG must include an ontological connection to the antibiotic molecule, mechanism of antibiotic resistance, and determinant of antibiotic resistance branches of the ARO, as outlined below. Notably, since our last publication the ARO terminology branch has been updated to include terms for phenotypic testing standards for the National Antimicrobial Resistance Monitoring System (NARMS) and National Committee for Clinical Laboratory Standards (NCCLS) to support clinical data harmonization efforts. Connections among terms within the ARO are supported by a set of relation terms from CARD’s Relations Ontology, some of which are generic (e.g. *is_a* or *part_of*) while others reflect key aspects of antimicrobial resistance (e.g. *confers_resistance_to_antibiotic*) (Table 1). With use of these relations, terms within the ARO form a knowledge network for AMR and combined with curation rules support computational approaches for annotation of genome sequences, interpretation of these annotations, and development of powerful curation quality control algorithms, as outlined below.

**Table 1.** Ontological relationships in use by CARD within the Antibiotic Resistance Ontology (ARO). New terms since the CARD 2020 publication are in bold

Relationship Label	Accession	Definition^d^
*is_a*	*n/a*	An axiomatic relationship wherein the subject class A is a subclass of class B. All instances of class A are also instances of class B.
*part_of*	BFO^a^:0000050	A relationship wherein a subject class A is but a part of class B. A core relation that holds between a part and its whole.
*has_part*	BFO:0000051	A relationship wherein a subject class A has a part class B (inverse of part_of).
*participates_in*	RO^b^:0000056	A relationship between continuant A and process B wherein A is somehow involved in B.
*confers_resistance_to_drug_class*	Pending^c^	A relationship wherein the subject class A confers or contributes to antibiotic resistance to drug class B.
*confers_resistance_to_antibiotic*	Pending^c^	A relationship wherein the subject class A confers or contributes to antibiotic resistance to antibiotic B.
*targeted_by*	Pending^c^	A relationship wherein molecule A is targeted by drug class B.
*targeted_by_antibiotic*	Pending^c^	A relationship wherein molecule A is targeted by antibiotic B.
*regulates*	RO:0002211	A relationships wherein the subject class A regulates the activity of class B.
*derives_from*	RO:0001000	A relationship between class A and class B wherein B inherits many properties from A.
*evolutionary_variant_of*	RO:0002312	A relationship wherein gene or protein A is a paralogous or orthologous variant of gene or protein B.
***is_small_molecule_inhibitor***	RO:0012006	A relationship between a continuant A and a process B, in which A is a small molecule that inhibits B.

^a^Basic Formal Ontology (http://purl.obolibrary.org/obo/bfo).

^b^Relations Ontology (http://purl.obolibrary.org/obo/ro).

^c^Custom relationships for CARD included in ARO but not yet in the official Relations Ontology.

^d^Paraphrased, see CARD website for full descriptive text.

In 2020, CARD v3.0.3 included 4336 terms from the ARO, up from 3567 in our 2017 publication (14), and covering 2923 AMR determinants encoded within CARD detection models (13). As of August 2022, CARD v3.2.4 has expanded to 6627 ARO terms and now includes 5057 AMR detection models covering 5010 reference sequences for resistance determinants (including 1933 curated resistance-associated variant mutations). While the ARO provides a detailed framework for describing antibiotics and AMR, CARD also applies ARO Classification Tags where curators manually ‘tag’ specific ARO terms as particularly informative for interpretation (13): primary tags AMR Gene Family, Drug Class, Resistance Mechanism, Antibiotic and secondary tags Efflux Component, Efflux Regulator, Adjuvant, Antibiotic + Adjuvant (Table 2). CARD’s secondary curation paradigm requires that ‘every curated AMR determinant must have an ontological path including each of the four primary ARO classification tags, i.e. the [AMR Gene Family] to which that determinant belongs, the [Resistance Mechanism], the [Drug Class] to which resistance is conferred, and the specific [Antibiotic] with a demonstrably elevated MIC’ (13). Overall, the curated sequence data in CARD v3.2.4 spans 458 AMR Gene Families (180 new since v3.0.3), 64 Drug Classes (15 new since v3.0.3), and 8 AMR Resistance Mechanisms (1 new since v.3.0.3) and the ARO contains additional information on 367 antimicrobial molecules and 33 adjuvants. Curation of impact of individual ARGs upon individual antibiotics via the *confers_resistance_to_antibiotic* relationship is ongoing (3386 curated to date from published MIC data), but CARD is also actively developing a MIC module to curate the quantitative MIC values reported in the scientific literature.

**Table 2.** ARO classification tags used to provide easy interpretation of genome annotations

Classification tag	Requirement^a^	Annotated ARO terms	ARO example^b^
AMR Gene Family	Primary	458	NDM ß-lactamase (ARO:300057)
Drug Class	Primary	64	Aminoglycoside (ARO:0000016)
Resistance Mechanism	Primary	8	Modification to cell morphology (ARO:3007115)^c^
Antibiotic	Primary	367	Streptomycin (ARO:0000040)
Adjuvant	Secondary	33	Tazobactam (ARO:0000077)
Antibiotic + Adjuvant^c^	Secondary	20	Ceftolozane-Tazobactam (ARO:3004724)
Efflux Component	Secondary	2	Efflux pump complex or subunit (ARO:3000159)
Efflux Regulator	Secondary	1	Two-component regulatory system modulating efflux (ARO:3000451)

^a^Primary tags are required for all CARD AMR determinants where applicable; secondary tags apply only rarely and can be omitted at the curator's discretion.

^b^Example names are abbreviated, see ARO accession in CARD for the complete description.

^c^New since CARD 2020 publication.

The ontology terms, detection models, and reference sequences in CARD v3.2.4 have all been sourced from 3004 publications, with the exception of many β-lactamases, as outlined below. Several core aspects of CARD have been reviewed, updated and/or corrected where necessary. These include: (i) updates to partial gene sequences, (ii) correction of translation frame errors for curated nucleotide sequences, (iii) taxonomic review and updates to over 40 pathogen species names (i.e. *Staphylococcus sciuri* updated to *Mammaliicoccussciuri*), (iv) systematic review of ARO term names, definitions, and synonyms, (v) review of the antibiotic molecule branch of the ARO with addition of disinfecting agents and antiseptics (and their ARGs where applicable) and (vi) review of the nomenclature of aminoglycoside acetyltransferases. Larger initiatives have also been undertaken by the CARD biocuration team and are described in more detail below.

### Updates to the Model Ontology

While CARD’s curated sequence data can be browsed, downloaded, or searched via BLAST at the CARD website, in our 2017 update (14) we introduced CARD’s Model Ontology, which allows AMR sequence and mutation reference data to be organized by the underlying specific mechanisms of resistance. These bioinformatics models are subsequently used by CARD’s RGI software to annotate genome or metagenome sequences. Descriptions of the model types and parameters in the MO have been updated to more accurately reflect their usage, with some removed due to their non-use. CARD currently curates molecular sequences under nine possible detection model types (each with a variety of parameters): protein homolog models (PHM), protein variant models (PVM), protein overexpression models (POM), rRNA gene variant models (RVM), protein knockout models (PKM), nonfunctional insertion models (NFI), gene cluster meta-models (GCM), efflux pump system meta-models (EPS), and protein domain meta-models (PDM). Supplementary Table S1 gives an overview of CARD’s MO-encoded detection model types and parameters.

Curation at CARD is routinely ahead of RGI software development, so not all MO parameters or models curated in CARD will be annotated in sequences analyzed using RGI. For example, RGI does not currently support the PKM, PDM, GCM or EPS model and meta-model types. In addition, while the PVM, POM and RVM model types are currently supported by RGI, mutation screening currently only supports annotation of resistance-conferring SNPs via the single resistance variant parameter. Supplementary Table S1 outlines all model types and parameters curated in CARD v.3.2.4, with indications of which are supported by RGI. Full descriptions of each model type and parameter in the Model Ontology are available at the CARD website and their use by RGI is outlined in the RGI documentation (https://github.com/arpcard/rgi).

### Ongoing cross-database harmonization of AMR determinants

A significant portion of AMR determinants in CARD represent β-lactamase (*bla*) genes or gene variants (∼71%). Among the 2124 determinants added since our 2020 publication, 1599 (75.3%) of these are described as β-lactam determinants. This is due, in part, to a large effort to coordinate and harmonize *bla* genes in CARD with those in the NCBI Pathogen Detection Reference Gene Catalog (15). NCBI is leading development of β-lactamase nomenclature rules and the naming of new β-lactamases (16), many of which lack corresponding published functional validation due to the rapid pace of sequencing-based discovery. As such, β-lactamases represent an exception to CARD’s primary curation paradigm: β-lactamase genes listed in the NCBI Reference Gene Catalog are included in CARD even if lacking experimental evidence of elevated MIC relative to a control or a peer-reviewed scientific publication. Overall, 72 new β-lactamase gene families have been added to CARD since 2020, along with general ontological improvements. For example, through review we discovered *bla*_ADC-68_, a published carbapenem-hydrolyzing class C β-lactamase (17) missing from CARD. This led to a restructuring of the *bla*_ADC_ branch of the ARO to differentiate *bla*_ADC_ genes with or without carbapenemase activity (see ARO:3005459), as well as the identification of several other missing *bla*_ADC_ lacking experimental examination of carbapenemase activity. Similarly, CARD curators performed literature reviews for β-lactamases with historically unclear nomenclature, such as the *Bacillus cereus* and *B. anthracis* BcI, BcII, BcIII, Bla1, and Bla2 class A and class B1 (metallo-) β-lactamases, harmonizing nomenclature with NCBI while updating the ARO to reflect the underlying complexity of function and evolutionary history. Yet, challenges continue for curation of unnamed, experimentally validated environmental β-lactamases discovered via functional metagenomics, such as found in wastewater, oil spills, and well water by Berglund *et al.* (18).

The mobile colistin resistance (MCR) phosphoethanolamine transferase (ARO:3004268) AMR gene family is another focus of ARO harmonization efforts. First detected in 2015 as MCR-1 (7), emergence of plasmid-mediated colistin resistance has spread and diversified rapidly (19) with over a hundred variants currently reported. MCR variants are grouped by gene family (MCR-1, MCR-2, etc.) with named individual alleles (20), similar to β-lactamase naming conventions. To reflect this in ARO, we expanded use of the semantic relationship *evolutionary_variant_of* (RO:0002312) which exists between MCR alleles of the same gene family (Table 1). For example, the MCR alleles MCR-1.2 (ARO:3004194), MCR-1.3 (ARO:3004514), and MCR-1.4 (ARO:3004515) are each described as *evolutionary_variant_of* MCR-1.1 (ARO:3003689). This is a relatively new addition to CARD’s Relationship Ontology, with the MCR gene family serving as a trial before potential further implementation for other AMR gene families, including particularly β-lactam resistance determinants, e.g. V240G variants of *bla*_KPC-3_ (21).

General cross-database harmonization efforts remain ongoing (both manual and software-assisted), with periodic database and literature surveys to identify problematic or absent ARO curation. A particular emphasis in the next year will be review of the current and historical nomenclature of aminoglycoside modifying enzymes, with an eye towards standardization.

### Integration of likelihood-based mutations for *Mycobacterium tuberculosis*

In 2020, CARD adopted novel model parameters for *Mycobacterium tuberculosis* ARG data as unlike other organisms examined, culture and antibiotic susceptibility testing for *M. tuberculosis* is very challenging. The TB community instead developed a likelihood framework, based on statistical association of SNPs with observed phenotypic resistance across a large number of sequenced genomes, which was collated in the Relational Sequencing TB Data Platform (ReSeqTB) (22). This database has since ceased operation, but fortunately CARD captured their last available data set from their 2019 Resistance Report totalling 424 mutations with significant likelihood scores for AMR. Resistance in *M. tuberculosis* is almost exclusively SNPs, but CARD could not use its Single Resistance Variant parameter from the Model Ontology as it is reserved for data with underlying experimental evidence of elevated MIC relative to controls. As such, we created new MO parameters based on the ReSeqTB framework to curate these data: no association with resistance TB (MO:0000053), indeterminate confidence TB (MO:0000054), minimal confidence TB (MO:0000052), moderate confidence TB (MO:0000051), and high confidence TB (MO:0000050) (Supplementary Table S1). These parameters are based on ReSeqTB’s likelihood ratio test (LR+) statistic that is used to evaluate whether mutations are positively or negatively associated with phenotypic resistance based on a drug susceptibility test, testing sensitivity, and specificity. Under the null hypothesis of no association, the LR value is expected to be 1, but deviations from this can be due to an association with resistance or a low number of available isolate samples. The LR+ measures the strength of association between the presence of a mutation and the drug resistance phenotype. For the purpose of CARD’s curation efforts mutations found to be non-significant (p > 0.05) were excluded, synonymous mutations were excluded, and mutations graded as no association with resistance or indeterminate have been archived but excluded from being displayed on the CARD website, included in download files, or RGI reporting. Future curation efforts will include incorporation of the large volume of information available in the recent CRyPTIC project (23,24).

### Expanded curation of resistance-modifying agents

One of the seven major branches of the ARO is resistance-modifying agents (ARO:0000076), representing antibiotic adjuvants and other molecules which modify AMR either directly, e.g. β-lactamase inhibitors, or indirectly through other cellular mechanisms, e.g. enhancing antibiotic entry or modifying cell physiology. Adjuvant molecules, as defined here, are not intrinsically growth-inhibitory on their own. This branch of the ARO was, however, historically given little attention and was not subject to definitive curation reviews. In our most recent update, we reviewed the existing ARO entries and the literature on adjuvants and other resistance modifying molecules, leading to significant ontological modifications, clarification of definitions, and expansion of included molecules. This led to re-organization of this branch of the ARO to include five novel ARO adjuvant categories: (i) inhibitor of antibiotic resistance mechanism (ARO:3007222), (ii) adjuvants enhancing antibiotic entry (ARO:3007223), (iii) adjuvants inhibiting antibiotic removal (ARO:3007224), (iv) adjuvants which alter physiology (ARO:3007225) and (v) host-related antibiotic adjuvants (ARO:3007226). In addition, β-lactamase inhibitors are now separated into serine β-lactamase inhibitor (ARO:3007128) and metallo-β-lactamase inhibitor (ARO:3007128) sub-branches, with further subcategorization based on chemical structure and composition, e.g. taniborbactam as a boronic acid β-lactamase inhibitor. Lastly, β-lactamase inhibitors are now tagged as Adjuvants using ARO Classification Tags (Table 2), with the newly added ontological relationship *is_small_molecule_inhibitor* (Table 1) used to encode the relationship between β-lactamase inhibitors and their target β-lactamase, e.g. tazobactam (a serine β-lactamase inhibitor) *is_small_molecule_inhibitor* of TEM-1, TEM-2, etc. While we acknowledge that antibiotics can act as adjuvants in clinically relevant antibiotic-antibiotic combinations (trimethoprim-sulfamethoxazole, quinupristin-dalfopristin, etc.), their intrinsic lethality on their own excludes them from the adjuvant definition presented herein.

## SUPPORTING MACHINE LEARNING WITH CARD SHORT NAMES

While some ARGs have established nomenclature rules, standardization of ARG names is not universal and some names curated into the ARO are very long. A common request from our data-focused users has been the addition of a curated and abbreviated naming convention for ARO vocabulary terms used for individual ARGs. Long ARO names are cumbersome or prohibited when dealing with commonly used data formats or software in bioinformatics and machine learning, e.g. *Escherichia coli gyrA conferring resistance to fluoroquinolones* (ARO:3003294). However, descriptive ARO names are also necessary for precluding inaccuracies and ambiguities among AMR determinants, particularly when managing point mutation-based resistance that often varies between taxa, e.g. the above term versus *Shigella flexneri gyrA conferring resistance to fluoroquinolones* (ARO:3003940). These two examples are necessarily descriptive, a requirement of ontologies, to both indicate the species from which the *gyrA* sequence was obtained and to differentiate point mutations specific to *E. coli* or *S. flexneri* which contribute to phenotypic fluoroquinolone resistance. Yet, at 63 and 64 characters these names are a bane for programmers, data scientists, and for concise visualization. To address this issue, we here introduce CARD Short Names, a CARD-specific abbreviation for ARG names associated with Antibiotic Resistance Ontology terms, often not based on the literature but instead designed by CARD curators.

If the original ARG name is 15 characters or less, the CARD short name is identical; if the gene name is >15 characters, the CARD Short Name has been abbreviated by CARD curators specifically to identify the proper gene or protein name. All CARD Short Names are unique and have whitespace characters replaced by underscore characters. The convention for pathogen names is capitalized first letter of the genus followed by the lowercase first three letters of the species name, for example ‘Abau’ for *Acinetobacter baumannii*. The antibiotic abbreviations are from abbreviations listed at the scientific journal *Antimicrobial Agents and Chemotherapy* (https://journals.asm.org/journal/aac/abbreviations) plus some custom abbreviations from the CARD curators, for example ‘AMG’ for aminoglycosides or ‘AZM’ for azithromycin. Simple CARD Short Names often do not involve either, e.g. CTX-M-15, but where applicable the CARD Short Names follow pathogen_gene or pathogen_gene_drug, e.g. *Ecol_gyrA_FLO* and *Sfle_gyrA_FLO* for the above examples. For ARGs conferring resistance to multiple drug classes, we use the generic term *MULT*, e.g. *MexEF-OprN with MexS mutations conferring resistance to chloramphenicol, ciprofloxacin*, and *trimethoprim* (ARO:3004068) becomes *Paer_MexS_MULT*, where ‘Paer*’* indicates the pathogen *Pseudomonas aeruginosa*. The full lists of abbreviations and CARD Short Names (5045 as of CARD v3.2.4) can be obtained at the CARD website (https://card.mcmaster.ca/download).

## ONLINE RESISTOME GENE IDENTIFIER (RGI) AND CARD:LIVE

**Figure 1.**
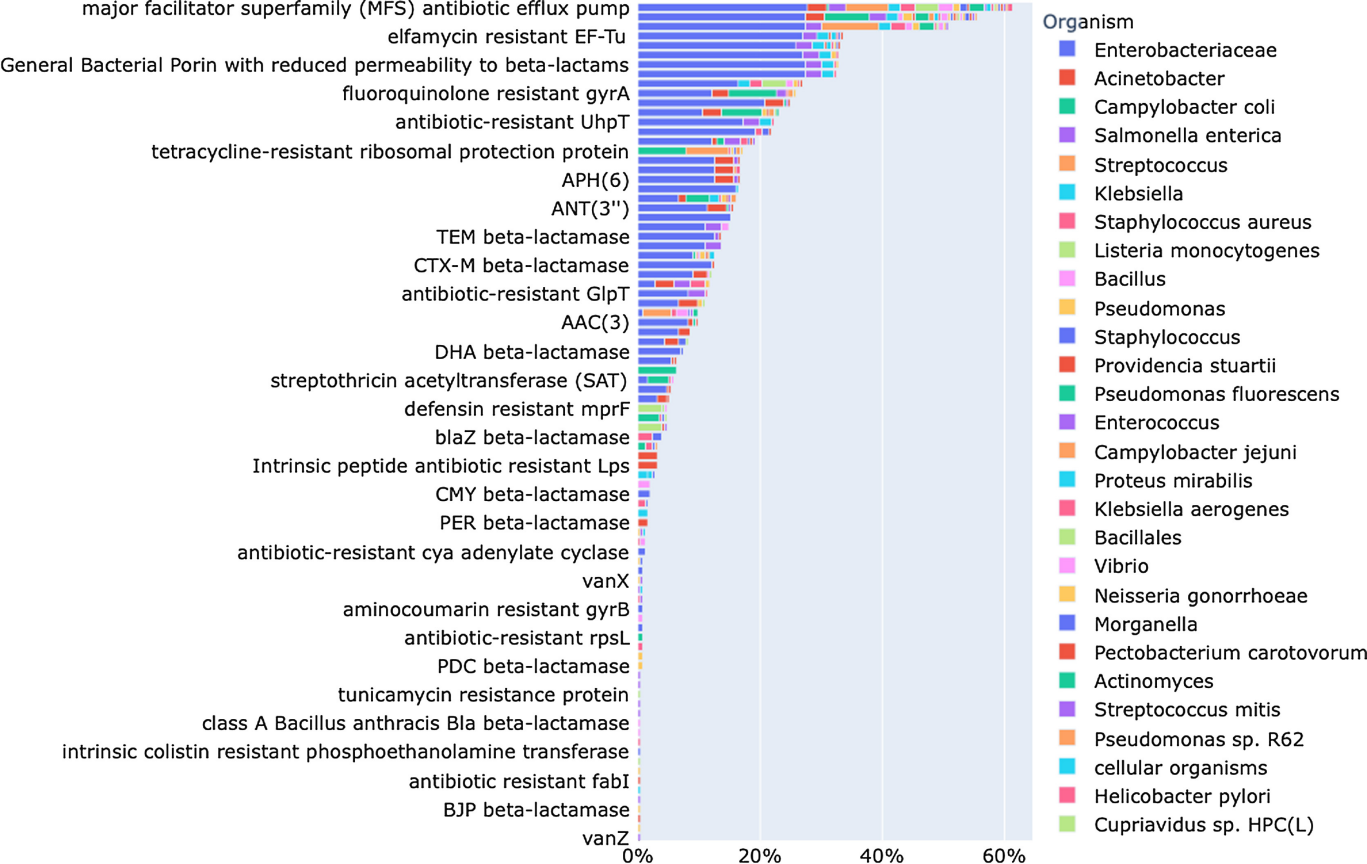
Prevalence of individual AMR Gene Families in data collected by CARD:Live, broken down by predicted pathogen of the submitted genome sequences. Approximately 60% of submitted genome sequences have an ARG from the major facilitator superfamily (MFS) antibiotic efflux pumps.

The CARD website allows users to predict ARGs from genome sequences using RGI, providing interactive visualization of the results organized by ARO Classification Tags and ARG annotation (https://card.mcmaster.ca/analyze/rgi). Usage of RGI is global and a genome sequence from a drug resistant infection is processed by RGI approximately every 4 min. In late 2020, we launched CARD:Live, an initiative to collect pathogen identification, MLST, annotated ARG names, date, and geographical region for genome sequences submitted to RGI online, providing a dynamic view of antibiotic resistant isolates from around the world being analyzed on RGI online (Figure 1). All information collected is voluntary, based on consent, and no sequence information is retained to protect the research programs of contributors. To date, over 13 000 RGI results have been collected and can be visualized online. Tools for versioning, downloading, or performing complex analyses of these data are forthcoming.

## *IN SILICO* PREDICTION OF CARD RESISTOMES AND VARIANTS

**Figure 2.**
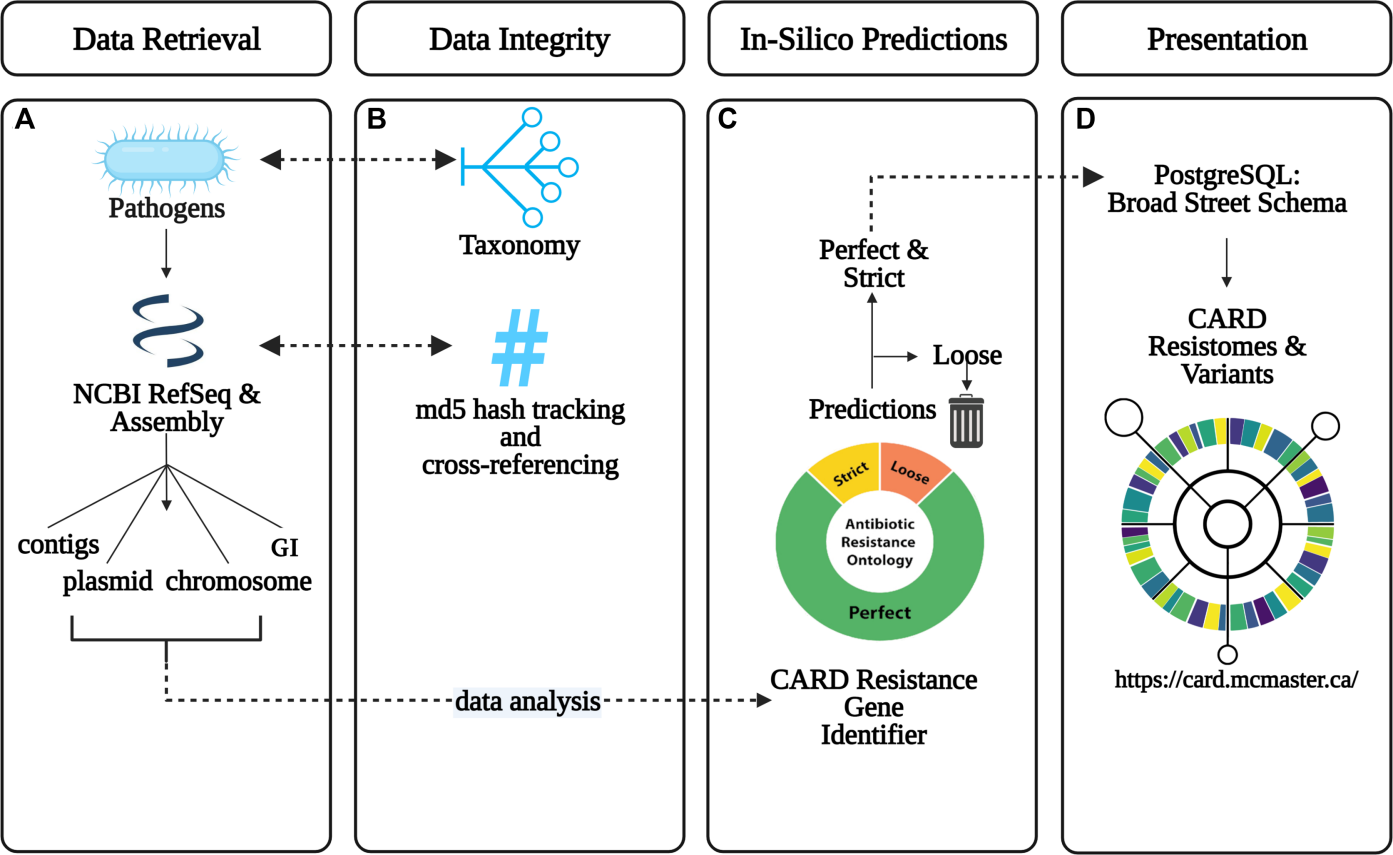
Schematic workflow for CARD Resistomes and Variants. (**A**) Data is retrieved by downloading available genome assemblies for a list of pathogens from NCBI’s RefSeq database. Retrieved genome assemblies are subsequently parsed to identify plasmid- and chromosome-originating sequences, while genomic islands are identified separately through IslandViewer 4. (**B**) Each pathogen is specified using the NCBI Taxonomy Database ID and the genome data integrity is verified with an MD5 checksum. (**C**) Each genome is analyzed with RGI to generate a predicted resistome, which is then filtered to remove divergent or otherwise dissimilar homologs below the respective CARD model threshold for RGI (‘Loose’). (**D**) The remaining high-fidelity predictions (RGI ‘Perfect’ and ‘Strict’ categories) are parsed to integrate predicted resistome information and genomic sequences into the Broad Street PostgreSQL schema underlying CARD, where the data is contextualized through CARD and the ARO. These data are then uploaded to the CARD website where they are available publicly for viewing, download, or use by RGI’s metagenomic read mapping algorithms.

In our 2020 publication, we introduced CARD Resistomes & Variants (13), a new database module for computationally predicted resistomes and ARG variant sequences to supplement canonical CARD curation of functionally characterized ARGs from the literature. Briefly, CARD-R uses CARD’s curated reference data, detection models, and RGI software to predict ARG sequence variants *in silico* from available genomic data for a targeted list of pathogens. This process thus predicts a putative resistome for each included genome or plasmid sequence, genomic island, or whole-genome shotgun assembly based on the AMR detection models curated in CARD and RGI’s ‘Perfect’ and ‘Strict’ annotations, where the ‘Perfect’ algorithm detects perfect matches to the curated reference sequences and mutations in CARD while the ‘Strict’ algorithm predicts variants of known ARGs, including screens for key mutations, using CARD’s curated bit-score cut-offs (Figure 2). At its inception, CARD-R consisted of a relatively small slate of pathogens (82 in total) chosen to encompass those of particular public health and research significance, e.g. the World Health Organization's (WHO) priority list of antibiotic resistant bacteria (2,25). Since then, CARD-R has expanded significantly, both in the volume of accumulated data (through additional pathogens and genomes) and the scale of collected AMR metadata. CARD-R v4.0.0 encompasses 377 pathogens covering 165 bacterial genera and 21,079 complete chromosomes, 41,828 complete plasmids, and 155,606 whole-genome shotgun assemblies from NCBI’s RefSeq catalog (26). An additional 50 bacterial pathogens are included in this *in silico* analysis, but lack prediction of any ‘Strict’ or ‘Perfect’ ARGs by RGI. A recent addition to CARD-R is the integration of 356,325 genomic island (GI) sequence predictions from IslandViewer 4 (27) for AMR determinant prediction, resulting in 2,662 GIs predicted to encode ARGs (across 1,084 genomes).

Overall CARD-R’s *in silico* prediction of resistomes yielded 322,710 unique nucleotide ARG allele sequences, each associated with ARO Classification Tags and covering 247 of CARD’s 461 AMR Gene Families and 31 of CARD’s 64 Drug Classes. Expansion of the list of pathogens included in CARD-R reflects harmonization with IslandViewer plus a targeted curation of pathogens associated with sepsis. The CARD-R data have several uses: (i) providing statistics on prevalence of individual ARGs within and among pathogens (e.g. *bla*_NDM-1_ is found in 47 pathogens), (ii) determining association of ARGs with mobile genetic elements as a step towards risk assessment, (iii) providing a more diverse reference sequence set for RGI’s metagenomic read alignment algorithms than available in canonical CARD (which is strongly biased towards common clinical pathogens due to its dependence upon the published literature) and (iv) providing data for RGI’s k-mer based ARG pathogen-of-origin prediction algorithms. Supplemental Table S2 details the breakdown of assemblies into chromosomal, plasmid, and assembly contig sequence origins and indicates the proportion of ARG-containing genomes for each pathogen.

## CONTINUOUS CURATION AND EXPANDED QUALITY CONTROL

As described previously, CARD has developed the custom ‘Broad Street’ schema for information storage and organization (13), broken down into six modules: controlled vocabularies, AMR reference sequences & detection models, resistomes & variants & prevalence; publications; external references; and administrative. Detailed description of this schema is now available online (https://github.com/arpcard/broadstreet_schema). The schema and data are managed with PostgreSQL 9.5.24 and the public CARD website and curator tools are designed with the Laravel 5.2.45 PHP framework, PHP 7.0.33, Apache 2.4.18 and PostgreSQL 9.5.24. The website, software, data, and curation issue tracking are all version-controlled using GitLab CE version 15.2.0.

CARD curation occurs continuously, with updates released every 1–3 months by a team of biocurators. Curation involves regular review of the scientific literature, supplemented by custom CARD*Shark text mining tools to identify high value publications in PubMED via machine learning. CARD curation generally starts with addition of a term for a new AMR gene. This will include entering the name of the gene, recording synonyms, writing a description, creating a novel CARD Short Name, and entering of the related publications. Curation may additionally include a Protein Data Bank structure (if available), private Curator Notes, or data for our Minimum Inhibitory Concentration module under development. If the ARO term instead describes an antibiotic molecule, we often curate a PubChem structure. We then create relationships between this term and the main branches of the ARO. This may include creating terms for new resistance mechanisms, antibiotics, or drug classes as well as adding ARO Classification Tags. With all the ontological and related information curated, the remaining major curation effort is defining the AMR gene detection model parameters for the gene, including reference sequences, mapped SNPs, bitscore cut-offs, definition of operons, or identification of all components of protein complexes.

One of CARD’s strengths is a dedication to expert, human curation of data. Yet, mistakes happen and there is a learning curve to accurately curating CARD for new staff. As such, CARD maintains a suite of Quality Control software tools (CARD-QC), which check a multitude of rules based on the Broad Street schema plus the ARO and other ontologies. These include checking text for use of whitespace, punctuation, and special characters; checking accuracy of citations from PubMED (including checking for updated citations), validating relations among ontology terms, including ARO’s curation paradigms, use of RO terms, and validation of synonyms; assessing direct and transitive (but not reflexive) closure for ontologies; check for missing curation of PDB or PubChem structures; ensure CARD Short Names are properly applied to all ARGs; validate ARO Classification Tags; ensuring the NCBITaxon ontology is taxonomically accurate and reflects any updated nomenclature; check reading frames, codons, and translation of reference sequences, including use of valid nucleotide or amino acid characters; check for updated sequences at NCBI; and checking completeness and values of model parameters based on detection model type.

## DATA AVAILABILITY, BAIT CAPTURE PROTOCOLS, AND COMMUNITY AMR CURATION

The CARD website (https://card.mcmaster.ca) provides tools for browsing and searching the ARO and other ontologies, names and descriptions of ARGs, CARD Resistomes & Variants & Prevalence data, the CARD:Live collection, tracking of changes for each release, plus laboratory protocols and bait sequences for performing ARG enrichment metagenomic sequencing based on CARD’s curated sequences (28). Online search tools include BLAST for comparing sequences to CARD reference sequences and the RGI for resistome annotation. CARD data can be downloaded online in a number of formats (TSV, OBO, OWL, JSON, FASTA). RGI software and documentation is available at GitHub (https://www.github.com/arpcard/rgi) and the ARO is additionally cross-posted to the Open Biomedical Ontologies’ OBO Foundry (http://purl.obolibrary.org/obo/aro). Updates to CARD are announced on Twitter (@arpcard) and via the CARD-L mailing list (see http://arpcard.mcmaster.ca/about). CARD curators are available to provide assistance via email to card@mcmaster.ca or posting of issues at CARD’s amr_curation GitHub repo (https://github.com/arpcard/amr_curation).

## CONCLUSIONS

In our 2020 update, we focused heavily on changes and improvements to the ontological structure underpinning CARD. Comparatively, only minor changes to the overall database schema and formatting behind the ARO are provided in this update. Instead, along with several quality improvements to general user experience and data access, as CARD continues to expand we have directed our efforts to more specialized and previously under-curated aspects of the ontology. This approach has led to large ontological and curation improvements for specific AMR gene families, drug classes, or individual branches of the ARO, as seen with the inclusion of over one-thousand additional β-lactamase sequences, the harmonization of MCR-based colistin resistance variants and nomenclature, the integration of likelihood-associated mutation parameters for *M. tuberculosis*, the addition of resistance to antiseptics, and the ongoing ontological overhaul of resistance-modifying agents. Additionally, greater effort has been expended towards integrating CARD with analytical software, including the significant expansion of CARD Resistomes & Variants, the introduction of CARD:Live, and development of the CARD Short Names standardized nomenclature. While CARD will continue to provide high-quality curated reference data, future improvements will expand from CARD’s focus upon individual ARGs to a pathogen-based holistic view of AMR prediction by combining the ARO with CARD-R as well as developing pathogen-specific curation and annotation rules.

## Supplementary Material

gkac920_Supplemental_Files

## Notes

Present address: Kara K. Tsang, Department of Infection Biology, London School of Hygiene and Tropical Medicine, London, United Kingdom.
